# Brain glymphatic fluid mapping in Alzheimer’s disease: a human MRI and PET study

**DOI:** 10.1093/braincomms/fcaf200

**Published:** 2025-05-23

**Authors:** Liangdong Zhou, Thanh D Nguyen, Gloria C Chiang, Samantha A Keil, Xiuyuan Hugh Wang, Tsung-Wei Hu, Haoyu Lan, Ke Xi, Ana Paula Costa, Emily B Tanzi, Lidia Glodzik, Jarek Wegiel, Tracy Butler, Mony J de Leon, Yi Li

**Affiliations:** Department of Radiology, Brain Health Imaging Institute (BHII), Weill Cornell Medicine, New York, NY 10065, USA; Department of Radiology, MRI Research Institute (MRIRI), Weill Cornell Medicine, New York, NY 10065, USA; Department of Radiology, Brain Health Imaging Institute (BHII), Weill Cornell Medicine, New York, NY 10065, USA; Department of Radiology, Division of Neuroradiology, Weill Cornell Medicine, New York-Presbyterian Hospital, New York, NY 10065, USA; Department of Radiology, Brain Health Imaging Institute (BHII), Weill Cornell Medicine, New York, NY 10065, USA; Department of Radiology, Brain Health Imaging Institute (BHII), Weill Cornell Medicine, New York, NY 10065, USA; Department of Radiology, Brain Health Imaging Institute (BHII), Weill Cornell Medicine, New York, NY 10065, USA; Laboratory of Neuro Imaging, USC Mark and Mary Stevens Neuroimaging and Informatics Institute, University of Southern California, Los Angeles, CA 90033, USA; Department of Radiology, Brain Health Imaging Institute (BHII), Weill Cornell Medicine, New York, NY 10065, USA; Department of Radiology, Brain Health Imaging Institute (BHII), Weill Cornell Medicine, New York, NY 10065, USA; Department of Radiology, Brain Health Imaging Institute (BHII), Weill Cornell Medicine, New York, NY 10065, USA; Department of Radiology, Brain Health Imaging Institute (BHII), Weill Cornell Medicine, New York, NY 10065, USA; Department of Developmental Neurobiology, New York State Institute for Basic Research in Developmental Disabilities, Staten Island, NY 10314, USA; Department of Radiology, Brain Health Imaging Institute (BHII), Weill Cornell Medicine, New York, NY 10065, USA; Department of Radiology, Brain Health Imaging Institute (BHII), Weill Cornell Medicine, New York, NY 10065, USA; Department of Radiology, Brain Health Imaging Institute (BHII), Weill Cornell Medicine, New York, NY 10065, USA

**Keywords:** parenchymal CSF mapping, glymphatic fluid volume, beta-amyloid, Alzheimer’s disease, magnetic resonance imaging (MRI)

## Abstract

Glymphatic system has been identified as a fluid exchange network in brain parenchymal for removal of toxic metabolites in Alzheimer’s disease. However, a clinically feasible, accurate, and non-invasive imaging technique for mapping global glymphatic fluid distribution throughout the entire brain and monitoring its dysfunction in Alzheimer’s disease is currently lacking. This cross-sectional retrospective study aims to compare three MRI-based measures of the glymphatic system structural alterations, a novel multi-echo T2 relaxometry-based parenchymal CSF (pCSF) mapping, T2 weighted-based segmented perivascular space burden, and diffusion tensor imaging-based free-water mapping. We evaluated their cross-correlation and investigated their respective association with PET measured beta-amyloid deposition using ^11^C-PiB PET. A total of 29 subjects (18 Female, age: 70.07 ± 8.73 years old), among them, 16 were cognitively normal and 13 were mild cognitive impairment or Alzheimer’s disease, underwent both MRI and ^11^C-PiB PET scans. Parenchymal CSF mapping, diffusion-based free-water mapping and perivascular spaces burden were generated from their respective MRI. Age and sex effects and group differences of these biomarkers were evaluated. The associations among these measures and Aβ deposition on ^11^C-PiB PET were analysed and compared. Our analysis demonstrated moderate correlations between pCSF, diffusion-based free-water mapping and perivascular space burden, suggesting these biomarkers capture overlapping yet distinct aspects of glymphatic fluid distribution. Importantly, linear regression analyses revealed that pCSF exhibited a significantly stronger positive association with beta-amyloid deposition (white matter: *t* = 3.536, *P* = 0.002, *R*^2^ = 0.446; Alzheimer’s disease-related meta regions: *t* = 4.510, *P* < 0.001, *R*^2^ = 0.541), compared with diffusion-based free-water mapping in white matter (*t* = 0.843, *P* = 0.407, *R*^2^ = 0.191) and perivascular space burden (*t* = 0.422, *P* = 0.677, *R*^2^ = 0.174), after adjusting for age and sex. This study identifies pCSF mapping, derived from multi-echo T2 data with clinically feasible 5 mins scan FAST-T2 sequence, as a potential non-invasive imaging biomarker for assessing glymphatic dysfunction in Alzheimer’s disease. The superior sensitivity of pCSF mapping arises from its direct quantification of glymphatic fluid at the water molecular level based on free CSF-specific T2 relaxation properties. These results suggest that pCSF could be useful in the monitoring of Alzheimer’s disease progression as beta-amyloid accumulating and predicting response to anti-amyloid therapies, potentially leading to better diagnostic strategies and therapeutic interventions of Alzheimer’s disease.

## Teaser

Zhou *et al*. reported that parenchymal CSF mapping (pCSF) derived from T2 relaxometry is more strongly associated with beta-amyloid deposition than other MRI-based glymphatic markers. Their findings suggest pCSF is a sensitive, whole brain, non-invasive biomarker for glymphatic dysfunction in Alzheimer’s disease, with potential utility in diagnosis and treatment monitoring.

## Graphical Abstract

**Graphical Abstract:**
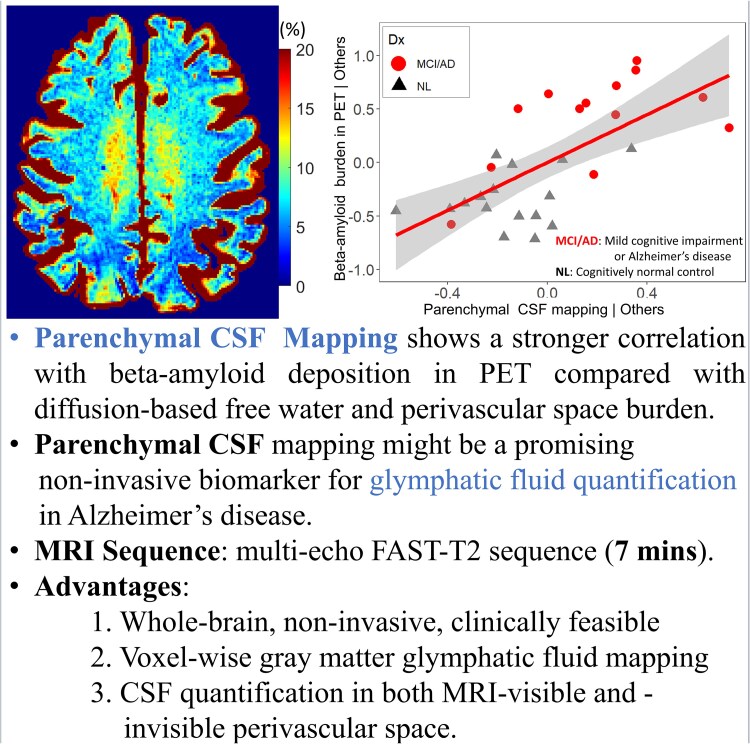

## Introduction

Glymphatic clearance, a recently characterized brain-wide mechanism for removing metabolic waste, including beta-amyloid (Aβ), has emerged as a key player in the pathogenesis of Alzheimer’s disease.^1-4^ This system relies on the perivascular spaces (PVS), which facilitate fluid exchange between cerebrospinal fluid (CSF) and interstitial fluid (ISF), enabling the transport and clearance of toxic metabolites. Disruption in glymphatic function is increasingly believed to contribute to Aβ accumulation, a hallmark of Alzheimer’s disease pathology.^1^ The dilation of PVS is thought to reflect CSF fluid stasis and reduced CSF fluid dynamics, implying glymphatic clearance dysfunction.^5,6^ Therefore, an accurate measure of the CSF fluid in PVS, termed as glymphatic fluid in this work, is important for evaluating the glymphatic clearance impairment and the progression of Alzheimer’s disease. However, accurately quantifying glymphatic fluid within PVS across the entire brain remains a significant challenge.

Several MRI-based techniques have been employed to assess the glymphatic pathway’s structure reflecting the glymphatic fluid volume information including PVS segmentation on T2-weighted imaging (T2w), diffusion MRI-based free-water imaging (DTI-FW),^7-9^ and recently emerged MRI relaxometry-based parenchymal CSF fraction mapping (pCSFF).^10-12^

The T2w-based PVS quantification focuses on the MRI-visible ones only. The typical approaches to quantifying PVS burden include a scoring system by counting the visible hyperintense spots on MR T2w,^13,14^ and the image processing-based segmentation of hyperintense spots on T2w in the cerebral white matter (WM).^15-17^ The scored PVS ratings has been shown that MRI-visible PVS in centrum semiovale is positively associated with brain amyloid in Alzheimer’s disease,^13^ and negatively associated with cognition.^14^ Yet, another MRI-visible PVS scoring study showed no association between PVS burden and PET measured amyloid load, suggesting the visible PVS could reflect underlying cerebral small vessel disease.^18^ Primarily, PVS extends beyond MRI-visible T2w-typerintense areas in WM, surrounding small blood vessels (arteriole, capillary and venule) in both WM and cerebral grey matter (GM).^18,19^ The PVS segmented in WM is therefore inherently an underestimation of total PVS and ignores the microscale PVS at the subvoxel level, which may result in an inaccurate estimation of glymphatic clearance dysfunction, weaken its association with the Aβ pathology, and delay the identification of subtle change of glymphatic fluid volume at early stage of Alzheimer’s disease.

The DTI-FW technique is the bi-tensor based model of free-water elimination in diffusion tensor imaging, which decomposes the total tissue water into anisotropic and isotropic diffusion components.^7-9^ In WM, the fibre tracts are well defined and the water molecule diffusion along fibre tracts is anisotropic, whose integrity can be quantified by the fractional anisotropy (FA).^20,21^ Accordingly, the water signal that is not being able to fit into anisotropic diffusion component is fitted as isotropic component and denoted as DTI-FW. Therefore, DTI-FW is generally considered a biomarker for extracellular fluids including ISF and CSF in PVS.^22,23^ DTI-FW has been shown to improve the accuracy and sensitivity of WM analysis in Alzheimer’s disease,^24^ and help to differentiate mild cognitive impairment (MCI) and Alzheimer’s disease from normal controls.^22,25^ Recently, higher DTI-FW in hippocampus has been associated with lower memory performance.^26^ However, there are limited researches on the association between DTI-FW and amyloid deposition in Alzheimer’s disease. One study has presented the association between MR-based parameters with amyloid but has not shown significant association between DTI-FW with amyloid deposition.^27^ These inconsistent results intrigue us to develop an accurate non-invasive measure of glymphatic fluid mapping.

Both MRI-visible PVS and DTI-FW lack specificity in capturing the glymphatic fluid in PVS and its association with Aβ deposition.^18,27^ To address the limitations aforementioned, we have developed a T2 relaxometry-based three-water-compartment model to measure parenchymal CSF fraction (pCSFF) using multi-echo FAST-T2 MRI by selectively filtering the signal that corresponds to water molecule with T2 between 200 and 2000 ms.^10-12^ In the model, the reconstruction result shows that the T2 of the CSF component is 2 s, which corresponds to the T2 value of free-water (CSF or glymphatic fluid in PVS) at 3T.^12^ pCSFF has been demonstrated to be associated with normal aging^11^ and with Alzheimer’s disease pathology^12^ in our prior publications. By calibrating pCSFF into an absolute fraction measure (pCSF), we aim to provide a more accurate representation of glymphatic fluid volume, facilitating comparisons with PVS burden (PVS/WMV). This technique, referred to as glymphatic fluid mapping, aims to provide a comprehensive view of glymphatic fluid dynamics across brain regions.

In this study, we calibrated parenchymal CSFF and DTI-FW into absolute fraction measures—pCSF mapping and FW mapping, respectively—using literature-reported total water content (TWC) values for GM and WM, assuming constant TWC based on prior findings.^28-31^ The human brain maintains a remarkably stable TWC across a range of physiological conditions. This stability has been confirmed in studies showing that even during dehydration, when the body undergoes significant changes in fluid balance.^29^ We used a constant TWC of 83% in GM and 70% in WM.^28-32^ This calibration allows for direct comparison between pCSF mapping, free-water mapping (FW) and the PVS burden normalized by WM volume (PVS/WMV). While each of these modalities offer insight into glymphatic system’s structure and function, they yield significantly different estimates of glymphatic fluid. Here we posit that pCSF provides the most accurate model for assessing glymphatic fluid in PVS throughout the brain. However, the comparison and association between PVS/WMV, pCSF and FW with Aβ deposition has not been reported.

We hypothesize that pCSF mapping, as a direct representation of glymphatic fluid, will demonstrate a stronger association with Aβ deposition than either FW or PVS/WMV. By establishing pCSF-based glymphatic fluid mapping as a sensitive and reliable biomarker, this study seeks to advance the monitoring of Alzheimer’s disease and predicting responses to treatment and support the development of therapeutic strategies targeting glymphatic clearance.

## Materials and methods

### Ethics approval

#### Consent to participate

All studies were approved by the WCM Institutional Review Board (IRB) and written informed consent was obtained from all participants.

#### Consent to publish

The authors affirm that human research participants provided informed consent for publication of the data collected.

### Participants

In this cross-sectional study, 29 subjects underwent MRI and ^11^C-PiB PET scans. All studies were approved by the IRB and written informed consent was obtained from all participants.

All subjects underwent standardized evaluations by a cognitive neurologist consisting of a clinical and neurological exam, interviews with subject and informant, Clinical Dementia Rating Scale, MOCA,^33^ the NACC Uniform Data Set V3.0 telephone cognitive battery,^34^ clinical blood tests, MRI and ^11^C-PiB PET. MRI and PET examinations were reviewed by a board-certified radiologist and all subjects were reviewed for final diagnosis in a multidisciplinary consensus conference and diagnosed as either NL or MCI/Alzheimer’s disease in accordance with NACC criteria. Subjects with MCI and negative amyloid on PET were excluded to ensure that all MCI are amyloid positive in Alzheimer’s disease spectrum.

### MRI image acquisition

MRI were acquired on a Siemens Prisma 3T scanner. The brain imaging protocol consisted of 3D MPRAGE T1w and T2SPACE sequences for anatomical structural imaging and PVS segmentation, multi-shell diffusion weight image (DWI) for mapping DTI-FW, as well as 3D FAST-T2 sequence for pCSF mapping, and T2 FLAIR sequence for WM hyperintensity (WMH) detection.^10^ The imaging parameters were as follows^11,35^: (i) 3D sagittal T1 MPRAGE: TR/TE/TI = 2300/2.3/900 ms, flip angle = 8°, readout bandwidth (rBW) = 200 Hz/pixel, voxel size = 1.0 mm isotropic, GRAPPA parallel imaging factor (*R*) = 2, scan time = 5.5 min, (ii) 3D sagittal T2w SPACE: TR/TE = 3200/408 ms, flip angle = 90°, rBW = 751 Hz/pixel, turbo factor = 285, voxel size = 1.0 mm isotropic; (iii) 3D axial FAST-T2 at two slice thickness: spiral TR/TE = 7.8/0.5 ms, nominal T2prep times = 0 (T2-prep turned off), 7.5, 17.5, 67.5, 147.5 and 307.5 ms, flip angle = 10°, rBW = 1042 Hz/pixel, number of spiral leaves per stack = 32, number of spiral leaves collected per T2prep = 64, voxel size = 1.3 × 1.3 × 2 mm^3^ (scan time = 7 min); (iv) 3D sagittal FLAIR SPACE with fat saturation: TR/TE/TI = 4000/384/2400 ms, echo spacing = 3.46 ms, flip angle = 90°, rBW = 751 Hz/pixel, turbo factor = 278, voxel size = 1.0 mm isotropic, *R* = 2, scan time = 5.4 min. DWI were acquired using a single shot, multiband, multi-shell sequence with both anterior–posterior and posterior–anterior phase-encoding, with TE/TR = 89.2/3230 ms, voxel size 2.5 mm isotropic, 98 directions and *b*-values = 1500, 3000 s/mm^2^.

### PET image acquisition

The ^11^C-PiB PET images were acquired using a Siemens Biograph mCT–S (64) slice PET/CT. The acquisition parameters were consistent with previous publications.^11,12,35^ The data was acquired in list mode from 40 to 90 min after rapid bolus injection of ∼555 MBq. PiB PET images were reconstructed to a 512 × 512 × 74 matrix of 0.8 × 0.8 × 3 mm voxels in 5 × 10 min time frames from 40 to 90 min with list mode.

### Image processing

#### MRI region of interest parcellation

Each T1w MRI was regionally segmented using FreeSurfer (FS)^36^ version 7.1 *recon-all* command for region of interest (ROI) parcellation. ROI for PiB Aβ SUVR quantification is a meta region in cortex and termed as Alzheimer’s disease cortical mask (ADmask).^3,12,37^ ROIs for pCSF and FW values included: WM and GM (only for pCSF).

#### 11C-PiB PET standard uptake value ratio

^11^C-PiB standard uptake value ratio (SUVR) was calculated using the cerebellar cortex as a reference region.^12,38^ Average SUVR within the cortical ADmask served as the overall measure of Aβ deposition.^37^ The Aβ status of subjects was also determined through a PET SUVR reading by two neuroradiologists (Y.L. and G. C.) both with more than 15 years of experiences.

#### pCSF mapping

Parenchymal CSF fraction map was obtained using non-linear least square fitting of the 3-exponential model with L2 regularization and consistent with previous reports.^10-12^ By enforcing constraint to each water component, pCSFF corresponds to the long T2 (200∼2000 ms) water that is freely movable, i.e. the CSF in PVS. It has been reported that the TWC is about 83% in human GM and 70% in WM.^28,31,32^ pCSFF can be converted to pCSF mapping by multiplying pCSFF with TWC = 83% in GM and TWC = 70% in WM to get an absolute measure of pCSF mapping in respective ROI. pCSF were then rigidly coregistered to FS T1w space using normalized mutual information criteria and the ROI values of pCSF were extracted by averaging the pCSF in all the voxels of the ROI. The validation to show the accuracy of pCSF mapping for CSF water by numerical experiment is available in Supplementary Fig. 1.

#### Diffusion tensor imaging-based free-water mapping

Diffusion MRI data were pre-processed using the human brain connectome pipeline, including geometric and eddy current distortions and inter-volume subject motion using the top-up and eddy toolboxes in FSL.^39-41^ DTI-FW was reconstructed using the bi-tensor free-water elimination model within the python DIPY package.^7,8,42^ The DTI-FW was converted to FW mapping in the same way as described for pCSF mapping above and coregistered to FS T1w space using the transform between FA to FS T1w. The ROI values of FWC were drawn from the ROI in FS T1w space. Only WM FWC is used in this study.

#### Perivascular spaces segmentation

PVS segmentation was performed using a deep learning-based model, which is a weakly supervised approach using Frangi filter segmented PVS as a prior.^16^ (Source code: https://github.com/Haoyulance/WPSS). Specifically, T1w was registered to T2w space and the enhanced PVS contrast (EPC) was derived from the T1w to T2w ratio.^15,16^ The PVS segmentation on EPC was constrained to WM region only. After excluding WMH from PVS mask, the total PVS volume was then normalized by WMV to obtain the PVS/WMV.

#### White matter hyperintensity segmentation

WMH were segmented using a transformer-based deep learning network, *wmh_seg*.^43^ (Source code: https://github.com/jinghangli98/wmh_seg). This network was trained on an unmatched dataset, including 1.5T, 3T and 7T FLAIR images from various sources, alongside artificially added MR artefacts. The WMH segmentation was visually checked for accuracy and minor manual edits were performed to add or remove small artefacts. The WMH mask was excluded from WM to minimize its effect on pCSF and FW in WM.

### Statistical analysis

Statistical analyses were performed in RStudio Version 2022.7. Shapiro–Wilk normality test was used to determine the appropriate statistical test models. The Spearman correlation between pCSF, FW and PVS/WMV was performed. The impact of covariates age, sex, APOE4 and diagnosis effects on the pCSF, FW and PVS/WMV was analysed using linear regression. Linear regression was also utilized to evaluate the association between pCSF, FW and PVS/WMV and the Aβ PET. A *P*-value < 0.05 was considered a significant effect. All *P*-values were corrected using FDR or Holm’s methods for multiple inference.^44^

## Results

### Participants

The details of the subject demographics and diagnostic values were summarized in Table 1. The last column *P*-values are corresponding to statistical tests of diagnostic group difference. A total of 29 subjects including 16 NL and 13 MCI/Alzheimer’s disease were recruited in this study. The MCI and Alzheimer’s disease were grouped together due to small sample size and easy match to NL. There were 12 participants with apolipoprotein 4ɛ (APOE4) in which 3 were NL and 9 were MCI/Alzheimer’s disease, showing a significant group difference by χ^2^ test and the increased risk of Alzheimer’s disease with APOE4 gene carriers. The *t*-tests showed a higher pCSF (WM: *P* = 0.001; GM: *P* = 0.003) in MCI/Alzheimer’s disease compared to NL. Wilcoxon rank-test showed higher PiB SUVR (W = 17, *P* < 0.001) in MCI/Alzheimer’s disease than NL.

**Table 1:** The subjects’ demographic and clinical information

Item	Overall	CN	MCI/Alzheimer’s disease	*P*-value
**Subjects number (*n*)**	29	16	13	
**Gender = Male (%)**	11 (37.90)	6 (37.5)	5 (38.5)	1
**Age, years (mean (SD))**	71.09 (9.20)	67.69 (8.40)	73.00 (8.54)	0.106
**APOE4 (*n*)**	12	3	9	0.018
**Aβ reading status = Positive (%)**	20 (68)	7 (44)	13 (100)	0.004
**ADmask PiB SUVR (mean (SD))**	1.57 (0.58)	1.17 (0.13)	2.07 (0.53)	<0.001
**WM pCSF, % (mean (SD))**	4.63 (0.37)	4.45 (0.29)	4.86 (0.34)	0.001
**GM pCSF, % (mean (SD))**	4.60 (0.39)	4.41 (0.31)	4.83 (0.36)	0.003
**WM FW, % (mean (SD))**	25.43 (1.17)	25.15 (1.26)	25.77 (0.99)	0.152
**PVS/WMV, % (mean (SD))**	1.59 (0.46)	1.52 (0.43)	1.68 (0.49)	0.373
**WMH, cm^3^ (mean (SD))**	4.34 (3.88)	3.36 (3.59)	5.54 (4.02)	0.092

WM, cerebral white matter; GM, cerebral grey matter; pCSF, parenchymal CSF content; FW, diffusion imaging tensor based free-water content; PVS, perivascular space volume; WMV, white matter volume; WMH, white matter hyperintensity.

*P* < 0.05 as significant level.

### Parenchymal CSF, free-water and perivascular spaces/white matter volume

**Figure 1:**
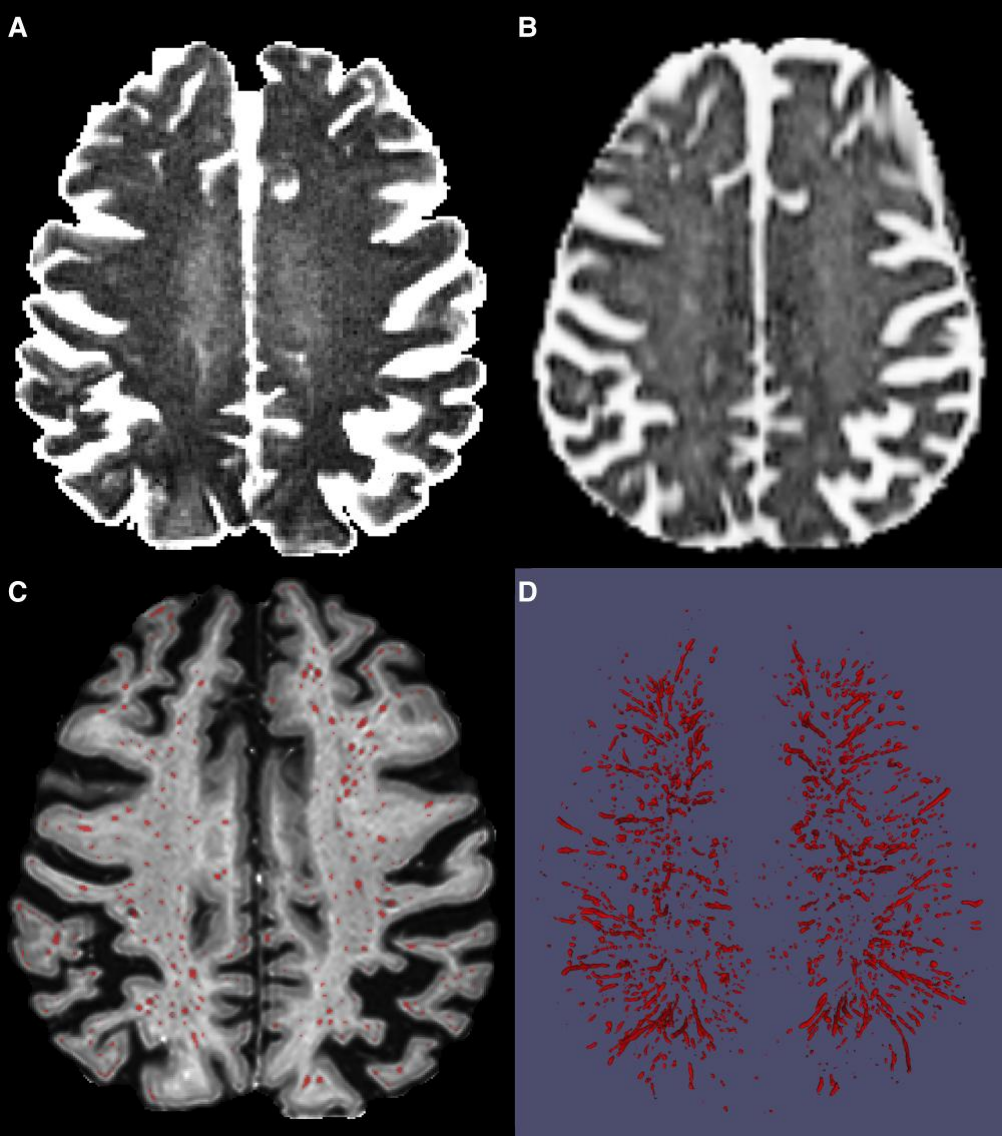
**Three types of potential glymphatic fluid volume measures.** (**A**) pCSF corresponding to long T2 signal in three water model fitting based on T2 relaxometry, range [0, 20%], i.e. light region represents higher pCSF value while dark regions indicate lower pCSF; (**B**) FW corresponding to isotropic diffusion water in bi-tensor DTI model, range [0, 100%] from dark to light; (**C**) binary PVS segmentation (red) overlayed on EPC map; (**D**) rendered whole brain 3D PVS volume. Of note the pCSF and FW are whole brain maps, but PVS segmentation is only feasible in WM on 3T MRI and its accuracy depends on image resolution and quality. pCSF, parenchymal CSF; DTI, diffusion tensor imaging; FW, DTI based free-water mapping; PVS, perivascular space; EPC, enhanced perivascular contrast.

Figure 1A-D showed a typical pCSF, FW, PVS segmentation over layed on EPC map and rendered 3D PVS structure for a 78-year-old female Alzheimer’s disease patient, respectively. Both pCSF and FW are whole brain maps, but PVS segmentation is only possible in WM.

### Correlation between parenchymal CSF, free-water and perivascular spaces/white matter volume

**Figure 2:**
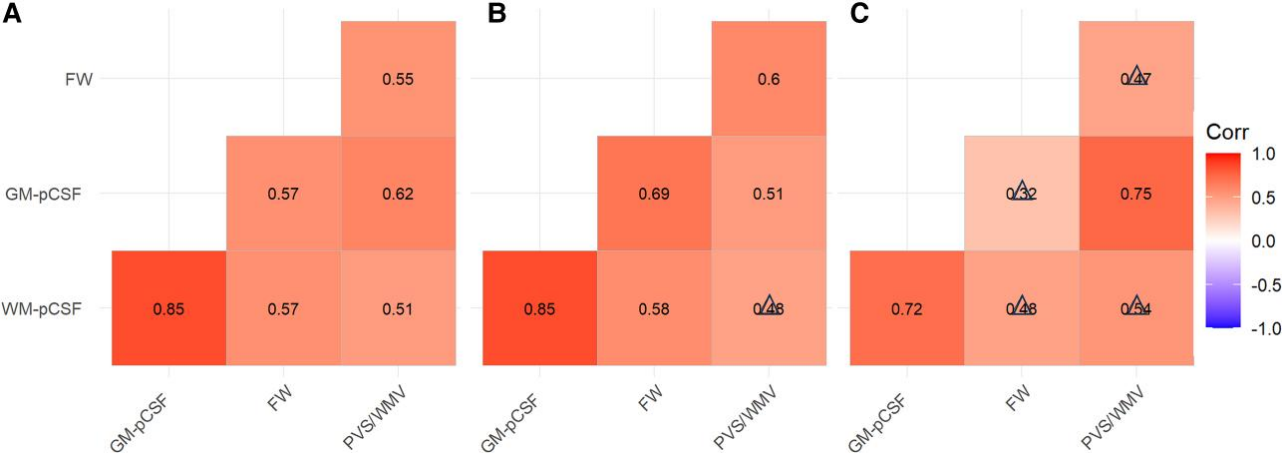
**The spearman correlations between pCSF, FW and PVS/WMV in WM.** (**A**) Spearman correlation map for all subjects (*n* = 29); (**B**) Spearman correlation map for NL group (*n* = 16); (**C**) Spearman correlation map for MCI/Alzheimer’s disease group (*n* = 13). Together, these show a higher correlation across biomarkers in NL than in the MCI/Alzheimer’s disease group, suggesting a potential inconsistency between these biomarkers when evaluating changes in glymphatic fluid when progressing from NL to MCI/Alzheimer’s disease. FW: Diffusion tensor imaging based free-water mapping; GM, grey matter; WM, white matter; pCSF, parenchymal cerebrospinal fluid mapping; PVS, perivascular space volume; WMV, white matter volume; PVS/WMV, perivascular space volume to white matter volume ratio; NL, cognitively normal control; MCI/Alzheimer’s disease, mild cognitive impairment or Alzheimer’s disease.

Figure 2 showed the mutual correlations between pCSF, FW and PVS/WMV. Figure 2A-C displayed the mutual correlation map between FW, GM pCSF, WM pCSF and PVS/WMV for the whole group, NL, and MCI/Alzheimer’s disease group, respectively. In the MCI/Alzheimer’s disease group, the correlations between these measures decrease compared with NL, implying increased inconsistency between pCSF, FW and PVS/WMV as Alzheimer’s disease pathology is introduced. The results show that these three biomarkers are significantly or tend to be positively correlated with each other either in all subjects or when restricted to clinical diagnostic group.

### Age, sex, apolipoprotein 4ɛ and diagnosis effects on parenchymal CSF, free-water and perivascular spaces/white matter volume

**Figure 3:**
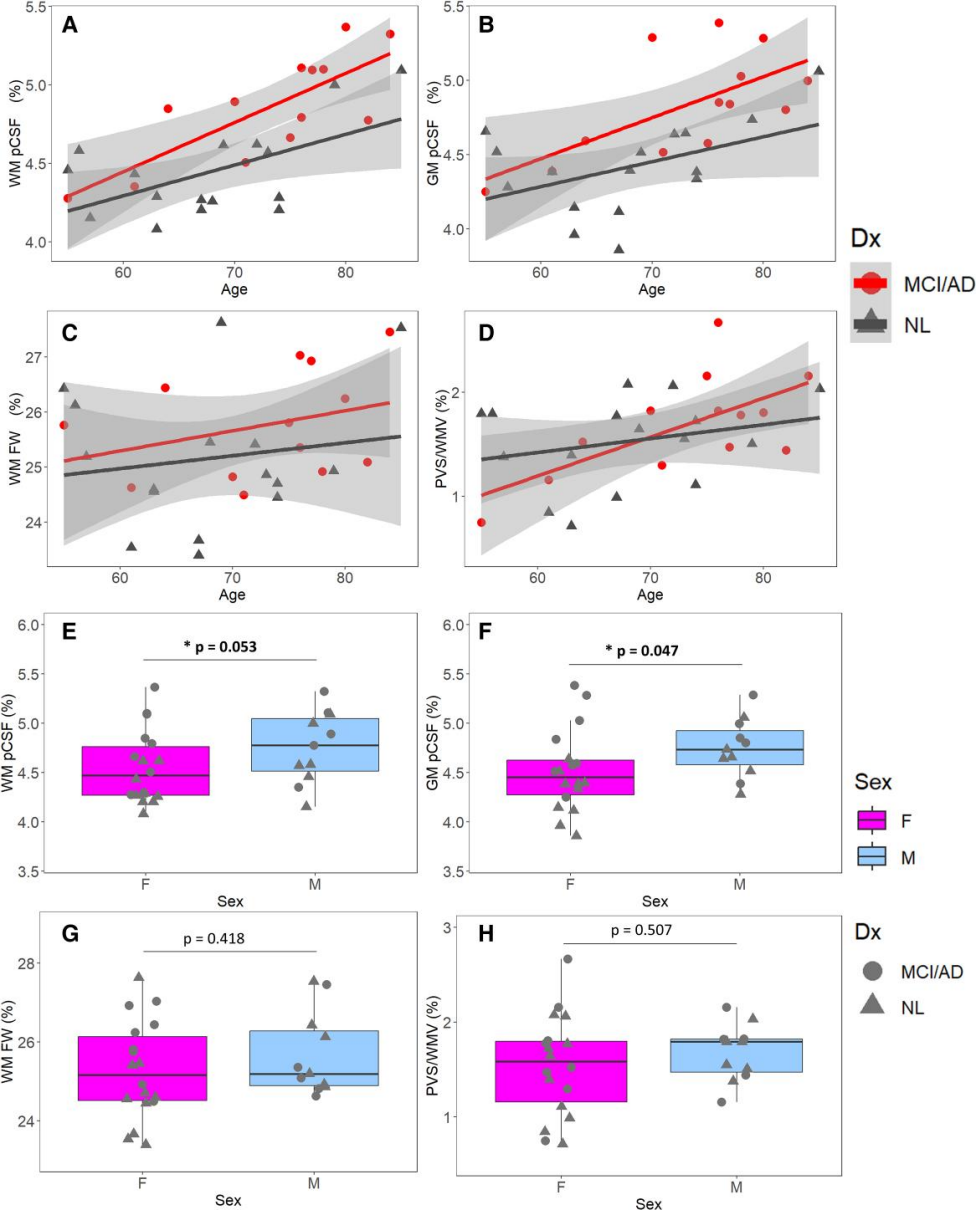
**The association of glymphatic fluid volume with age, sex and dx based on multi-variable linear regression (*n* = 29 includes 16 NL and 13 MCI/Alzheimer’s disease).** From (**A**-**H**) circles represent MCI/Alzheimer’s disease subjects and triangles indicate NL participants. (**A**-**D**) are WM pCSF, GM pCSF, FW and PVS/WMV with age grouped by Dx. Both WM and GM pCSF show an age, diagnosis and sex differences, showing the potential diagnostic value of pCSF in Alzheimer’s disease studies. FW shows no effects on age, sex and diagnostic group. PVS/WMV only shows age effect. (**E**-**H**) are WM pCSF, GM pCSF, FW and PVS/WMV by sex. Both WM and GM pCSF show a sex specific difference not observed in either FW or PVS/WMV. FW, diffusion tensor imaging based free-water mapping; GM, grey matter; WM, white matter; pCSF, parenchymal cerebrospinal fluid mapping; PVS, perivascular space volume; WMV, white matter volume; PVS/WMV, perivascular space volume to white matter volume ratio; NL, cognitively normal control; MCI/Alzheimer’s disease, mild cognitive impairment or Alzheimer’s disease.

In a linear regression model with age, sex, APOE4 and clinical diagnosis as independent variables and pCSF/FW/PVS as the dependent variable, i.e. pCSF/FW/PVVS ∼ age + sex + Dx + APOE4, it showed that APOE4 status is not significant for all models. After removing APOE4 status as a covariate, the model pCSF/FW/PVVS ∼ age + sex + Dx demonstrated a strong *R*^2^ = 0.632 and indicated that older subjects exhibited increased WM pCSF (age: *t* = 4.704, *P* < 0.001), and those with MCI/Alzheimer’s disease expressed a higher WM pCSF (DxNL: *t* = −3.145, *P* < 0.01) in Fig. 3A. Additionally, males expressed a marginally higher WM pCSF (sexM: *t* = 2.033, *P* = 0.053) in Fig. 3E. In GM, this same linear regression model found similar results for pCSF demonstrating an *R*^2^ = 0.537 and an increased GM pCSF with age (age: *t* = 3.481, *P* < 0.01) and MCI/Alzheimer’s disease (DxNL: *t* = −2.945, *P* < 0.01) in Fig. 3B. Similarly, an increased GM pCSF is observed in males (sexM: *t* = 2.089, *P* = 0.047) in Fig. 3F. Overall, pCSF in both GM and WM showed age, diagnostic and sex difference, offering a promising clinical value of pCSF in Alzheimer’s disease studies.

For DTI-FW, the same model showed a low *R*^2^ = 0.035. Specifically, FW showed no significant relationship with diagnostic group (DxNL: *t* = −1.027, *P* = 0.314) or age (age: *t* = 1.073, *P* = 0.294) in Fig. 3C and no sex differences (sexM: *t* = 0.823, *P* = 0.418) in Fig. 3G.

For PVS/WMV, the analysis yielded a *R*^2^ = 0.149, and a significant increase of PVW/WMV with age (age: *t* = 2.490, *P* < 0.05), but not with diagnostic group (DxNL: *t* = −0.179, *P* = 0.859) in Fig. 3D. Similarly, there was no sex difference of PVS/WMV (sexM: *t* = 0.674, *P* = 0.507) in Fig. 3H.

### Association of parenchymal CSF, free-water and perivascular spaces/white matter volume with beta-amyloid deposition

**Figure 4:**
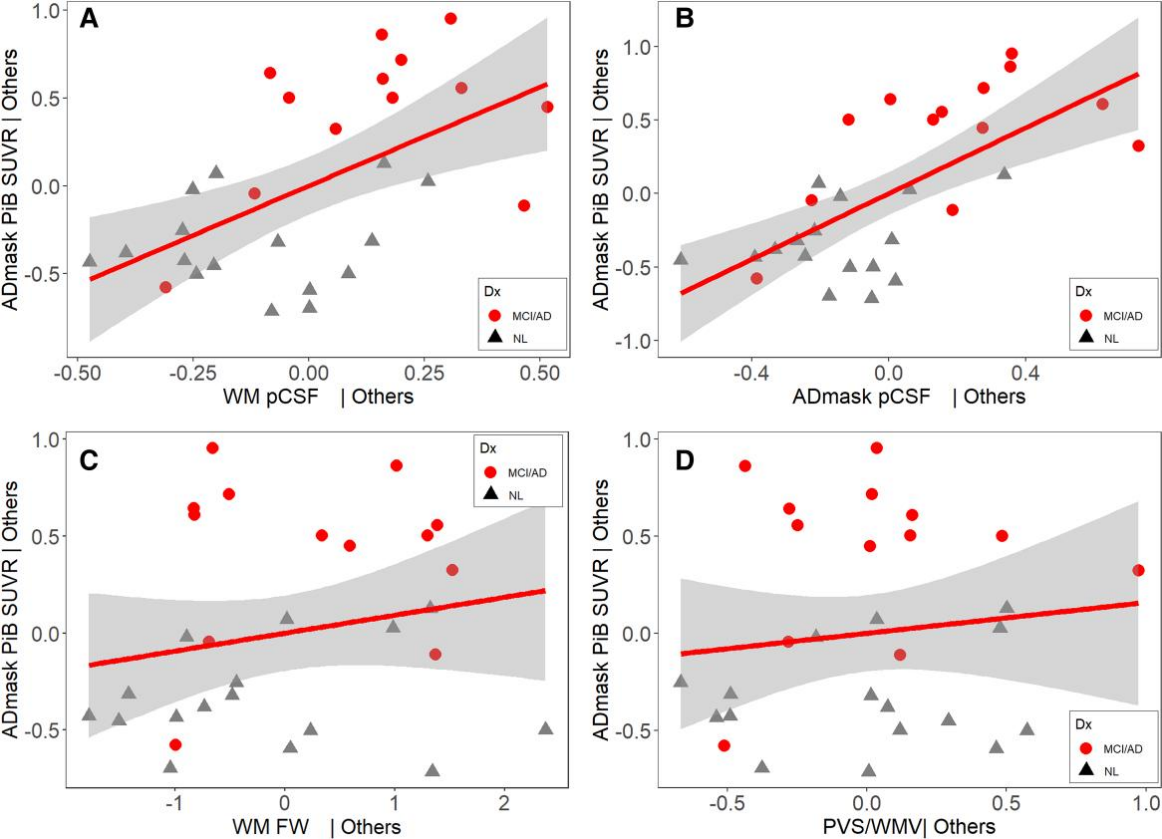
**The partial regression plots between glymphatic fluid volumes and PiB SUVR controlling for age and sex in linear regression model (*n* = 29).** Red circles represent MCI/Alzheimer’s disease subjects and black triangles indicate NL. (**A**) ADmask PiB SUVR versus WM pCSF; (**B**) ADmask PiB SUVR versus ADmask pCSF; (**C**) ADmask PiB SUVR versus FW; (**D**) ADmask PiB SUVR versus PVS/WMV. The results showed that PiB SUVR was positively associated with pCSF in both GM and WM, but was not associated with FW and PVC/WMV ratio, indicating a superior performance of pCSF when associated with beta-amyloid deposition, an Alzheimer’s disease hallmark pathology. FW, diffusion tensor imaging based free-water mapping; GM, grey matter; WM, white matter; ADmask, meta region in grey matter for Alzheimer’s disease-related beta-amyloid deposition; pCSF, parenchymal cerebrospinal fluid mapping; PVS, perivascular space volume; WMV, white matter volume; PVS/WMV, perivascular space volume to white matter volume ratio; SUVR, standard uptake value ratio; NL, cognitively normal control; MCI/Alzheimer’s disease, mild cognitive impairment or Alzheimer’s disease.

In the linear regression with age, sex and WM pCSF as independent variables, and ADmask PiB SUVR as dependent variable, i.e. SUVR_ADmask ∼ pCSF_WM + age + sex, the results demonstrated a moderate *R*^2^ = 0.446. To make the visualization of the relationship straightforward, we used partial regression plot between pCSF and PiB SUVR in ADmask, adjusted for age and sex. For DTI-FW and PVS/WM, the similar models and plots were applied below. PiB SUVR was positively associated with WM pCSF (*t* = 3.536, *P* = 0.002) as shown in Fig. 4A. There was a lower PiB SUVR observed in male subjects (*t* = −2.267, *P* = 0.032), and no significant association between age and PiB SUVR was observed (*t* = −0.027, *P* = 0.944). The similar model for ADmask pCSF, a slightly better results was achieved with adjusted *R*^2^ = 0.541. ADmask pCSF, very close to GM pCSF, was positively associated with PiB SUVR (*t* = 4.510, *P* < 0.001) as shown in Fig. 4B as partial regression plot. Males again exhibited a lower PiB SUVR (*t* = −2.340, *P* = 0.024), and no age effects were observed (*t* = −0.097, *P* = 0.923) for PiB SUVR.

Comparatively when this linear regression model was performed with DTI-FW, as shown in Fig. 4C, the model performed poorly with an *R*^2^ = 0.191, displaying no significant association of DTI-FW with PiB SUVR (*t* = 0.843, *P* = 0.407). There was a significant association of age (*t* = 2.528, *P* = 0.018) and no sex effects (*t* = −1.109, *P* = 0.278) for PiB SUVR were observed.

Furthermore, for PVS/WMV, as presented in Fig. 4D, the model (*R*^2^ = 0.174) showed PiB SUVR was significantly increased with age (*t* = 2.306, *P* = 0.029), but was not associated with sex (*t* = −1.026, *P* = 0.315) and PVS/WMV (*t* = 0.422, *P* = 0.677).

## Discussion

This study presents pCSF mapping as a novel and promising biomarker for assessing parenchymal glymphatic fluid distribution and its alterations in Alzheimer’s disease. Our findings demonstrate that pCSF mapping, derived from a T2 relaxometry-based three-water-compartment model, provides a more accurate representation of glymphatic fluid volume compared to traditional methods such as DTI-FW mapping and image segmentation-based PVS burden estimation. The term glymphatic fluid mapping emphasizes the functional role of pCSF mapping as a measure of glymphatic clearance efficiency. Unlike PVS burden, which often underestimates glymphatic fluid by excluding subvoxel-level structures and PVS within GM, and DTI-FW, which overestimates it by including ISF, pCSF mapping directly targets the specific glymphatic fluid component.^11,12^ By calibrating pCSFF into absolute fraction measures (pCSF), we achieved a more comprehensive representation of glymphatic fluid dynamics across brain regions, enabling a robust comparison with Aβ deposition.

### Role of perivascular spaces in glymphatic clearance

PVS serve as key pathways for glymphatic clearance, facilitating the removal of metabolic waste, including Aβ, from parenchyma.^1,45^ However, these structures are often not visible with conventional neuroimaging techniques,^46^ limiting our understanding of drainage-system pathology at the microscopic level.^18^ By utilizing pCSF mapping to quantify glymphatic fluid, we are able to overcome some of these limitations and gain a more comprehensive understanding of glymphatic function and dysfunction.^12,46^

Our findings corroborate the hypothesis that dilation of the PVS, reflects glymphatic fluid stasis and implies impaired glymphatic function—a contributing factor to the pathogenesis of Alzheimer’s disease.^35,47^ Traditional methods of quantifying PVS burden may not fully capture the entirety of the PVS network, especially subvoxel-level structures in both WM and GM.^13,15^ This underestimation potentially obscures the true extent of glymphatic dysfunction.

Our focus on pCSF mapping as a measure of glymphatic fluid volume in PVS further highlights the importance of advanced imaging techniques in assessing the efficiency of this clearance pathway. The findings suggest that pCSF provides a more direct measure of glymphatic fluid volume and clearance efficiency due to its specific sensitivity to long T2 water components associated with glymphatic fluid.^10,48^ Numerical experiments and cross-correlation analyses support the validity of pCSF as an accurate estimation of glymphatic fluid within the PVS. Our data underscore the potential for targeted interventions that might enhance this system’s function, offering new avenues for preventing or slowing the progression of Alzheimer’s disease.^49^

### CSF in perivascular spaces as glymphatic fluid

Despite increasing attention on the glymphatic system, a major challenge in the field remains the accurate characterization and quantification of glymphatic fluid. This fluid, residing in PVS, plays a central role in waste clearance, yet the literature inconsistently refers to it using terms such as ‘CSF-like water’,^50,51^ ‘ISF’^52,53^ or simply ‘CSF’.^45,54^ To unify our discussion and emphasize the biological role of this fluid in clearance pathways distinct from subarachnoid CSF,^55^ we refer to it as ‘glymphatic fluid’ in this work. While this nomenclature is not yet standardized, it underscores the fluid’s functional significance in metabolite removal and its potential contribution to Alzheimer’s disease pathology. The increase of glymphatic fluid volume might be associated with the stagnant or stasis of glymphatic fluid,^27^ which further implicates the impairment of glymphatic function.^56,57^

### Glymphatic fluid mapping: correlations among MRI measures and association with amyloid deposition

Our study provides key insights into the relationships among three MRI-based measures of glymphatic system structural alterations —pCSF,^11^ DTI-FW^7,22,25^ and PVS burden^18^—as well as their associations with beta-amyloid (Aβ) deposition in Alzheimer’s disease. The moderate correlations observed among pCSF, DTI-FW and PVS burden suggest that all three measures capture overlapping aspects of glymphatic fluid dynamics,^2,52^ thereby reinforcing their validity as imaging biomarkers of glymphatic system pathology.

However, when examining patient subgroups, we found that correlations among these measures weakened in individuals with MCI or Alzheimer’s disease compared to cognitively normal (NL) controls. This attenuation of correlations in disease states underscores the increased variability and complexity of glymphatic dysfunction as Alzheimer’s disease progresses. While both pCSF and DTI-FW reflect changes in fluid compartments within the brain, their specificity differs. DTI-FW likely overestimates glymphatic fluid by including ISF,^7^ which may incorporate fluid changes associated not only with glymphatic dysfunction but also neuroinflammatory processes or oedema. Whereas pCSF provides a more direct measure tied to the long T2 water components associated with glymphatic fluid in PVS.^11^ Notably, from NL to MCI/Alzheimer’s disease, mean pCSF increased by ∼9.5%, similar to the PVS segmentation increase of 10.5%, whereas FW only increased by about 2.5% (Table 1). This highlights pCSF’s greater sensitivity to disease-related changes.

Our findings elucidate the intricate relationship between glymphatic clearance mechanisms and Alzheimer’s disease pathology, particularly beta-amyloid (Aβ) deposition.^4,47,58^ Among the three metrics studied—pCSF, DTI-FW and PVS/WMV—pCSF exhibited the strongest association with Aβ deposition. This superior correlation underscores the unique sensitivity of pCSF mapping in capturing the glymphatic system’s role in the clearance of neurotoxic proteins, reinforcing its potential as a precise and clinically relevant biomarker. pCSF’s robust correlation with Aβ deposition in both GM and WM emphasizes its ability to reflect glymphatic dysfunction across the brain. The higher *R*² values observed for GM pCSF compared to WM pCSF suggest that glymphatic activity in GM regions may play a more significant role in Aβ clearance, possibly due to the higher metabolic demand and waste production in these regions.^12^ In contrast, PVS burden, which often fails to detect subvoxel-level PVS or fully represent fluid in GM areas, showed limited association with Aβ deposition.

Glymphatic fluid stasis, as reflected by increased pCSF, likely impairs the clearance of toxic metabolites, contributing to Aβ accumulation and disease progression. By isolating the glymphatic fluid component within PVS, pCSF mapping advances our understanding of the glymphatic system’s efficiency and its relationship with Aβ deposition. Unlike traditional methods, which may overestimate or underestimate glymphatic fluid due to their broader or narrower scopes, pCSF mapping captures subtle, physiologically relevant changes in glymphatic system pathology.

The integration of pCSF mapping into glymphatic fluid mapping not only reinforces the validity of this approach but also highlights its clinical potential. The superior sensitivity of pCSF mapping indicates that it could significantly enhance the monitoring of Alzheimer’s disease progression and predicting of responses to treatments, potentially leading to better diagnostic strategies and therapeutic interventions targeting glymphatic clearance pathways.^59,60^ By offering a precise and dynamic representation of glymphatic fluid, pCSF mapping bridges the gap between structural and functional imaging, providing novel insights into mechanisms of neurodegeneration that may not be detectable with conventional methods.

### Age, sex, apolipoprotein 4ɛ and diagnosis effects of parenchymal CSF, free-water and perivascular spaces/white matter volume

The investigation into the effects of age, sex and diagnosis (NL versus MCI/Alzheimer’s disease) on pCSF, FW and PVS/WMV revealed significant variability across different demographic and pathological groups.

#### Age effects

Age significantly influences pCSF, underscoring the natural age-related changes in glymphatic clearance and CSF dynamics.^11^ This supports the hypothesis that age-related decline in glymphatic efficiency potentially contributes to the accumulation of neurotoxic substances like amyloid-beta.^12^

#### Sex effects

Sex exhibited a marginal effect on pCSF in WM and significant effect in GM. This suggests that there may be a difference in glymphatic mechanisms between sexes, potentially influenced by biological or hormonal factors. APOE4 effects: APOE4 status is not a significant factor for pCSF, FW and PVS/WM in the linear model, suggesting that this genetic factor may not have a direct effect on the morphological distribution of the glymphatic fluid pathway. However, previous studies have shown that APOE4 is associated with pathological changes or reduced efficiency across multiple brain homeostatic pathways, including glymphatic clearance.^61,62^ In this study, there was no significant difference in pCSF between APOE4 carriers and non-carriers (two sample *t*-test, *t* = −1.40, *P* = 0.18), which may be attributed to the small sample size and imbalanced APOE4 distribution across diagnostic groups. A more definitive conclusion regarding the relationship between APOE4 and pCSF will require a larger, well-powered study.

#### Diagnosis effects

The diagnostic status revealed a pronounced difference in pCSF, with MCI/Alzheimer’s disease subjects exhibiting higher pCSF, reinforcing the association between impaired glymphatic clearance and neurodegenerative processes characteristic of Alzheimer’s disease.^63^ Comparative Sensitivity of pCSF, FW and PVS/WMV: Conversely, both FW and PVS/WMV showed weaker associations with age and diagnostic status, indicating their limited sensitivity to the glymphatic system’s changes associated with aging and Alzheimer’s disease pathology. These results highlight the superior sensitivity of pCSF as a biomarker for detecting alterations in glymphatic clearance related to aging and neurodegeneration, suggesting its potential utility in early diagnosis and monitoring of Alzheimer’s disease progression.^59^

#### Summary of findings

Overall, the findings demonstrate that pCSF is more responsive to demographic and pathological changes compared to FW and PVS/WMV. The greater sensitivity of pCSF makes it a more reliable biomarker for assessing glymphatic dysfunction, which is closely linked to the pathogenesis of Alzheimer’s disease. In contrast, FW and PVS/WMV may provide complementary information but are less effective in capturing the nuanced changes in glymphatic clearance associated with disease progression.

### Other potential MRI-based glymphatic dynamics measures

While the biomarkers introduced in this report, pCSF, DTI-FW and PVS burden potentially offer morphological information of the glymphatic pathway, mainly depicting the distribution of PVS-related glymphatic fluid, there are also many other emerging techniques that aim at delineating the dynamic or functional properties of glymphatic system.^63-65^ DTI along the PVS (DTI-ALPS) is a diffusion imaging-based method that quantifies the ISF diffusivity along the PVS of medullary veins at the level of lateral ventricle.^63^ The limitation of DTI-ALPS is that it is measured as an index from a local region in WM, which might lose the regional variation of neurofluids dynamics and to what extend it can capture glymphatic function is not validated.^35,66-68^ Grey matter and CSF coupling is one topic that studies the interaction between brain activities and CSF dynamics using functional MRI (fMRI).^64,69,70^ This approach aims to study the involvement of sleep- or activities-dependent brain activities on the brain clearance or glymphatic function, which could be useful to help understanding how the decreased CSF dynamics interact with PVS enlargement or vice versa. Another approach using dynamic diffusion weighted imaging (dynDWI) to study the vacular pulsatility and CSF coupling.^71,72^ This method quantifies the CSF dynamics in periaterial CSF pulsatility of the pial arteries and studies its pattern and coupling with vascular dynamics, trying to understand the cardiac pulsation-driven glymphatic fluid behaviour in healthy and patients.^54,65^ The morphological and functional properties are two main aspects of glymphatic function/fluid studies. Currently, there is no one model or sequence that captures the full information of glymphatic function. Using both morphological (pCSF, DTI-FW and PVS) and functional (DTI-ALPS, fMRI, and dynDWI) data with various MRI sequences will help us to obtain a comprehensive and complementary information of the glymphatic function and guide the related therapeutic development in Alzheimer’s disease.^3,4,73-75^

### Limitations of this study

We would like to acknowledge several limitations of this study. Firstly, the cross-sectional nature of this study limits our ability to draw causal inferences between glymphatic dysfunction and Alzheimer’s disease progression. Additionally, our sample size, though adequate for preliminary findings, may not capture the nuanced effects of glymphatic clearance across different stages of the disease.^76^ The diagnosis groups MCI and Alzheimer’s disease were merged together as MCI/Alzheimer’s disease to have easy compare with NL group in terms of number participants. The use of MCI/Alzheimer’s disease group ignored the significant cognitive variability within this group, which might contribute to the glymphatic fluid distribution and affect the analysis model. The cognitive scores or disease severity could be added as a confounding factors in the future analysis with larger sample size. Furthermore, pCSF mapping has not yet been validated in animal models. Future work should focus on the validation in animal model using two-photon imaging or similar microscale imaging techniques.^77^ By addressing these challenges, pCSF mapping holds promise to significantly enhance our understanding of glymphatic clearance and its role in neurodegenerative diseases, ultimately guiding novel interventions to mitigate Alzheimer’s disease progression.

### Conclusion

Our study establishes pCSF mapping as a sensitive and clinically feasible biomarker for evaluating glymphatic fluid distribution in Alzheimer’s disease. By demonstrating a stronger association with beta-amyloid deposition compared to traditional methods, pCSF-based glymphatic fluid mapping emerges as a superior tool for detecting glymphatic dysfunction and its contribution to Alzheimer’s disease pathology. The ability of pCSF mapping to isolate glymphatic fluid from other compartments, such as ISF, underscores its precision and potential for advancing the monitoring of Alzheimer’s disease and predicting responses to treatments. These findings provide a foundation for integrating glymphatic fluid mapping into diagnostic and therapeutic frameworks, offering new opportunities to target glymphatic dysfunction as a modifiable factor in Alzheimer’s disease progression.

## Supplementary Material

fcaf200_Supplementary_Data

## Data Availability

Raw data were generated at the Brain Health Imaging Institute (BHII) at Weill Cornell Medicine (WCM). Derived data and code for analysis supporting the findings of this study are available upon reasonable request from the corresponding authors. The code for PVS segmentation was available: https://github.com/Haoyulance/WPSS. The code for WMH segmentation was available at https://github.com/jinghangli98/wmh_seg.
