# Supplementary material for: Brain glymphatic fluid mapping in Alzheimer’s disease: a human MRI and PET study
Source: Brain Commun. 2025 May 23;7(3):fcaf200. doi: 10.1093/braincomms/fcaf200 (PMC12130685; doi:10.1093/braincomms/fcaf200)
Supplement: fcaf200_Supplementary_Data [file fcaf200_supplementary_data.docx]

# Supplementary material

## Numerical and phantom experiments for pCSF validation

### Numerical experiments

We numerically simulated the fitting of pCSF from three compartment model and tested the accuracy of pCSF compared with the ground truth. Specifically, we simulated the total T2 signal as $y=a*e^{-\frac{t}{T_{2,csff}}}+b*e^{-\frac{t}{T_{2,mwf}}}+c*e^{-\frac{t}{T_{2,iewf}}}+\epsilon$, where $a+b+c=1$ and $\epsilon$ is added Gaussian noise. $T_{2,csff}$ was set 2000 ms, $T_{2,iewf}$ was 70 ms, and $T_{2,mwf}$ was 10 ms, as ground truth of T2 relaxation time of each water component. The added noise level was to make sure the simulated signal had SNR=150, which is similar or lower than as the real human data. The time $t$ was set 0.5, 8, 18, 68, 148, 308, 1008 ms, and $a=5\%, b=10\%, c=85\%$ are the ground truth fractions of three water components, respectively. Then the simulation was performed 1000 times, and the mean and standard deviation of error were computed.

### Numerical experiments results

The 1000 times repeats of numerical simulation showed that the mean of reconstructed pCSF was 4.88%, mean of error in pCSF was 0.12% with a standard deviation of 0.72%, indicating that most fittings gave results less than 1% error. The mean of relative error was 2.4%. The distribution of the reconstructed results was reported as shown in the Boxplot in Figure S1. The paired t-test shows that the error is significantly different from zero (t = 0.99, p < 0.001). However, the error is considered acceptable given the low simulated SNR, which is lower than the typical real data acquired in human. Since the mean error is very close to zero, we are safe to conclude that the ROI averaged pCSF has no bias compared with the ground truth.


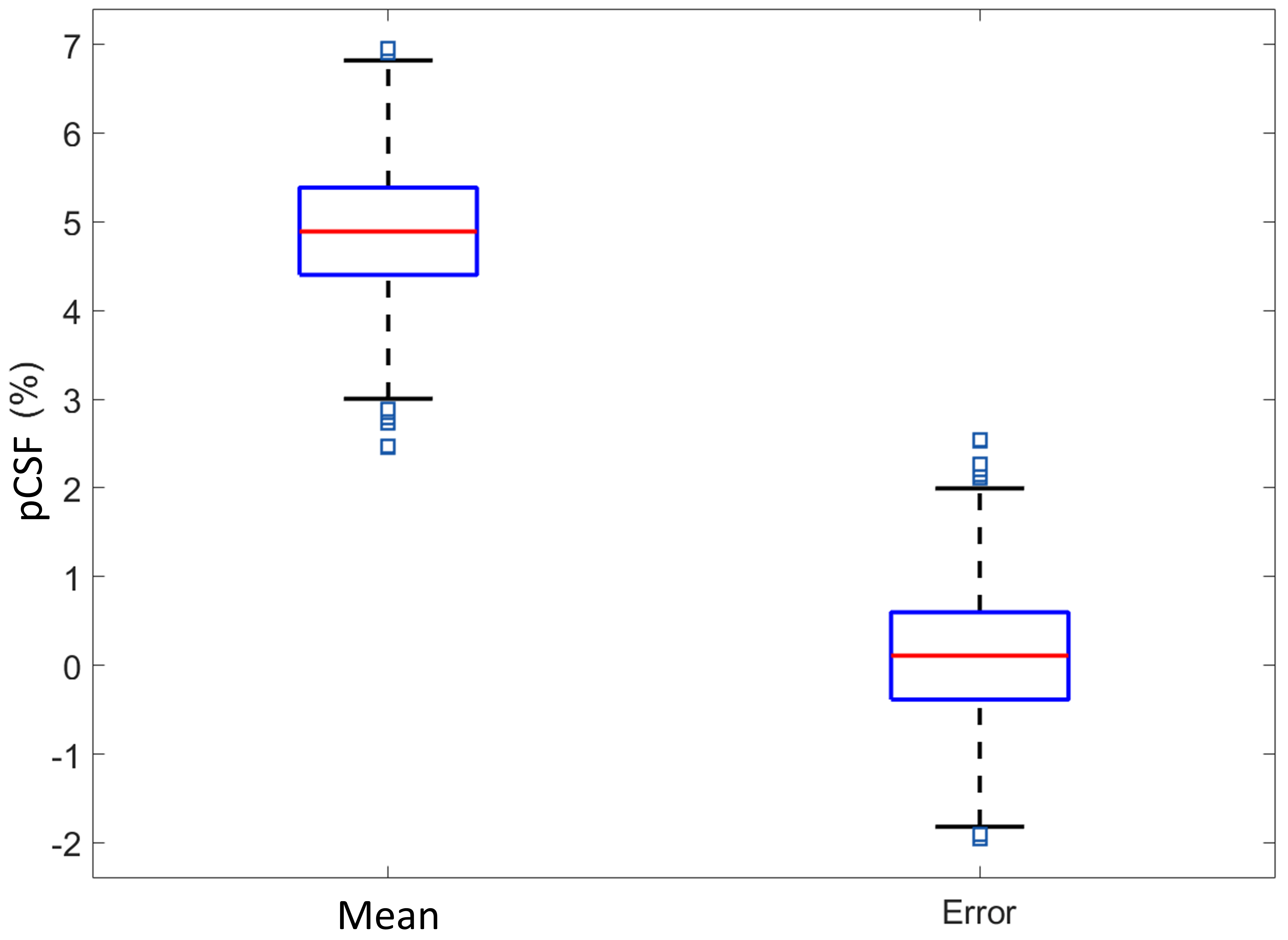


**Supplementary Figure 1**. pCSF error in numerical simulated reconstruction with n=1000 samples. It showed the mean of reconstructed pCSF is 4.88%, which is very close to the ground truth of 5% and the mean of error is 0.12%. The paired t-test shows that the error is significantly different from zero (t = 0.99, p < 0.001). However, the error is considered acceptable given the low simulated SNR, which is lower than the typical real data acquired in human.

## What is pCSF measuring?

Based on the reconstruction of pCSF from multi-echo T2 data, the pCSF is corresponding to the water that has long T2 time (T2 > 200 ms). The pure water in 3T MR scanner has been reported to have T2 time about 2 seconds. As the water molecule becomes more freely movable from constricted environment, the T2 time of the molecule becomes longer. We enforced a constraint of T2 time (T2 is in [200 ms, 2000 ms]) during the construction to make sure the pCSF is corresponding to long T2 component in the total signal.^1^ The water in brain tissue is composite of intracellular fluid, ISF, CSF and blood. The water in intracellular space and interstitial space are mostly restricted and has relatively short T2 time (< 200 ms). The T2 time of water in blood has been reported around 186 ms, which is less but close to our T2 cutoff for pCSF.^2^ This indicates that our measured pCSF could be dominated by CSF in PVS and a little bit contaminated by the blood water.

## Supplementary references

1. Zhou L, Li Y, Sweeney EM, et al. Association of brain tissue cerebrospinal fluid fraction with age in healthy cognitively normal adults. *Front Aging Neurosci*. 2023;Vol. 15(1162001). doi:https://doi.org/10.3389/fnagi.2023.1162001

2. Chen JJ, Pike GB. Human whole blood T2 relaxometry at 3 Tesla. *Magnetic Resonance in Medicine*. 2009;61(2):249-254. doi:10.1002/mrm.21858
